# Effects of a Long-Acting Formulation of Octreotide on Patients with Portal Hypertension

**DOI:** 10.1155/2017/3943210

**Published:** 2017-08-10

**Authors:** Pei-Jing Cui, Jing Yao, Yin Zhu, Zheng-Yun Zhang, Jun Yang

**Affiliations:** ^1^Department of Geriatrics, Ruijin Hospital, School of Medicine, Shanghai Jiao Tong University, Shanghai 200233, China; ^2^Department of Surgery, Shanghai Jiao Tong University Affiliated Sixth People's Hospital, Shanghai, China

## Abstract

**Objective:**

This study aimed to determine whether the treatment of a long-acting formulation of octreotide (OCT-LAR) exerted a similar effect on improving the prognosis of patients with portal hypertension compared with placement of transjugular intrahepatic portosystemic shunts (TIPSs).

**Methods:**

A total of 24 patients with portal hypertension who underwent TIPS placement or OCT-LAR treatment from January 2010 to January 2015 were reviewed. Hemodynamic studies, biological values, live functions, and treatment complications before and during the treatment were evaluated.

**Results:**

Baseline clinical characteristics were similar between two groups. Hepatic venous pressure gradient (HVPG) was improved in OCT-LAR groups (15.9 ± 2.4 to 12.8 ± 1.6 mmHg). Both groups showed a slight decrease in endothelin-1 (ET-1) and urotensin II and a slight increase in oxide metabolite (NO*x*) concentrations with no significant difference. Aspartate aminotransferase and alanine aminotransferase increased one week after TIPS placement when they improved in the OCT-LAR treatment group. The complications of OCT-LAR treatment were minor and transient. However, one patient who received TIPS placement presented procedure-related complications and required rehospitalization, and 2 patients had developed hepatic encephalopathy during the follow-up period.

**Conclusion:**

Prolonged administration of OCT-LAR exerted a virtually similar effect on improving hemodynamic parameters and liver function in patients with portal hypertension compared with placement of TIPS, with no apparent serious adverse effects.

## 1. Introduction

Portal hypertension (PH) usually results from an initial increase in intrahepatic resistance with a secondary increase in portal venous blood flow. When the pressure gradient between the portal vein and the inferior vena cava exceeds a threshold of 10 mmHg, clinical consequences including variceal bleeding, ascites, and hepatorenal syndrome become significant [1–3]. PH-related variceal bleeding remains a life-threatening condition with a mortality rate of approximately 20%. Rebleeding from the varices occurs in about 60% of patients at one to two years after the initial bleeding episode, with a mortality rate that exceeded 30% [4, 5]. Prevention of variceal rebleeding remains a significant challenge. Usually, the medical treatment aim is to reduce portal pressure and/or decrease blood flow in portosystemic collaterals to diminish the risk of rebleeding. Transjugular intrahepatic portosystemic shunts (TIPSs) or pharmacological therapies, such as somatostatin analogues, have usually been regarded as the preferred therapeutic strategy for this purpose. TIPS was introduced in the 1980s and has been regarded as a major technical advancement in the management of PH-related complications [6]. The goal of TIPS placement is to reduce portal pressure by shunting blood from the portal to the systemic circulation, bypassing the liver. Currently, TIPS is overwhelmingly preferred over the traditional surgical shunts because of its less invasive technique and faster recovery time [7]. However, complications occur during or after the TIPS placement procedures, including intraperitoneal hemorrhage, portal vein perforation, and hepatic artery or bile duct injury, influencing its clinical use [8]. Octreotide (OCT), one of the somatostatin analogues, acts by inhibiting glucagon and blunting the postprandial increase in hepatic venous pressure gradient (HVPG) and portal blood flow [9]. Its effects in controlling variceal bleeding virtually without side effects had been proved by clinical studies and meta-analysis [10–12]. However, the current treatment regimen of subcutaneous injections three times daily is not ideal for long-term drug administration in cirrhotic patients. Hence, the introduction of OCT-LAR has made chronic treatment possible. Following a single intramuscular injection, OCT-LAR concentrations reach a plateau at day 14 and remain relatively constant for the following 3 to 4 weeks in adults [13]. A small randomized controlled study showed that after 3 months of therapy, cirrhotic patients who received 20 mg of OCT-LAR intramuscularly every month presented a statistically significant reduction in HVPG [14]. However, the conclusion of the trial is controversial because only 18 patients were included in the trial; thus, the trial provided insufficient evidence of the efficiency profile of OCT-LAR in the cirrhotic population.

Therefore, a retrospective review of the medical records of patients who accepted TIPS placement or OCT-LAR administration after initial bleeding controlling was carried out. The purpose of this study is to decide whether the treatment of OCT-LAR exerts a similar effect in improving the prognosis with fewer complications in patients with PH compared with TIPS.

## 2. Methods

### 2.1. Patients

The records of patients at Shanghai Jiao Tong University Affiliated Sixth People's Hospital and Ruijing Hospital with PH who accepted TIPS placement or OCT-LAR administration after initial bleeding control from January 2010 to January 2015 were reviewed. All patients with initial bleeding were treated by endoscopic band ligation at once and received systematic and prolonged use of beta blockers afterwards. A beta blocker (nonselective) was given, and the dose was adjusted to reduce baseline heart rate by 25% or to reach a pulse rate of 55/min. 72 hours after the bleeding control, the patients were evaluated and either TIPS or LAR was given. Demographic variables, hemodynamic studies, biological values, and adverse event data before and after the treatment were collected. The diagnosis of PH was confirmed by aspiration biopsy or was made based on computed tomography (CT) and/or magnetic resonance imaging (MRI). The degree of hepatic dysfunction was graded according to the criteria of Child and Turcotte [15]. The side effects of TIPS placement or OCT-LAR during the follow-up period were recorded.

### 2.2. Treatment Procedure

TIPS placement was performed according to previously described methods [16] in the interventional radiology unit. After direct portography between the portal and hepatic veins, the shunt tracts were lined with various commercial stents: Wallstents (Boston Scientific Corp, Quincy, MA) and others. If necessary, balloon dilation was performed to reduce the pressure gradient. The portal systemic pressure gradient was 7–10 mmHg in the TIPS procedure performed by our medical team.

All patients were evaluated by the same medical team according to the follow-up schedule. Doppler duplex ultrasonography (US) was performed 24 h and 1, 3, and 6 mo after TIPS procedure, followed by every 6 mo thereafter or whenever recurrent TIPS dysfunction was suspected clinically. TIPS dysfunction was suspected on the US if the intrastent flow velocity was less than 60 cm/s or higher than 120 cm/s or if there was a change in the direction of the flow in the intrahepatic portal branches compared with previous US findings. TIPS dysfunction was defined as a shunt narrowing of more than 50%, a portosystemic pressure gradient higher than 12 mmHg or both. Primary patency was defined as the interval of time without an intervention. A systematic invasion stent dilatation was conducted in the presence of suspected dysfunction [16].

A single dose of 20 mg OCT-LAR (Novartis Pharma, Basel, Switzerland) was intramuscularly administrated once a month for patients who accepted pharmaceutical treatment.

### 2.3. Hemodynamic Studies

Hepatic hemodynamics was performed according to the recommended standard [17]. As previously described [18], an 8-F curve catheter (Cordis Europa, Amsterdam, Netherlands) was carefully introduced under fluoroscopy into the right main hepatic vein. The external zero reference was set at midchest. The wedged hepatic venous pressure (WHVP) was measured when the tip of the catheter was wedged in a small hepatic vein, providing a stable pressure tracing with fine venous fluctuations during a minimum of 30 s. To verify that the catheter was in a proper wedged position, contrast media was also injected to visualize liver parenchyma and small portal branches. The free hepatic venous pressure (FHVP) was measured when the catheter floated in the hepatic vein near the junction with the vena cava. HVPG, which is an estimate of portal pressure [19], results from the difference between the WHVP and FHVP.

### 2.4. Assessment of Biological Values

The hepatic venous blood was sampled during hepatic hemodynamic studies. Samples taken in heparinized tubes were transported immediately on ice to the laboratory. After centrifugation for 15 min at 5°C, aliquots were prepared under pyrogen-free conditions and kept frozen at −70°C until assay. The serum concentrations of ET-1 and urotensin II (URO II) were measured by commercially available enzyme-linked immunosorbent assay (ELISA) kits according to the manufacturer's guidelines. The plasma concentration of oxide metabolites (nitrite and nitrate as NO*x*) was measured using a specific fluorometric assay (Cayman Chemical Company, Ann Arbor, MI).

Peripheral blood samples were analyzed for aspartate aminotransferase (AST), alanine aminotransferase (ALT), total bilirubin (TB), and prothrombin time (PT) by using standard laboratory methods.

### 2.5. Statistics

Results are expressed as mean and standard error of the mean (SEM), and all statistical analyses were performed using the Statview 5.0 Program (Abacus Concepts, Berkeley, CA). A *P* value of less than 0.05 was considered statistically significant.

## 3. Results

### 3.1. Characteristics of Patients

The two groups of patients were well matched in sex, age, severity of liver dysfunction, cause of liver disease, and follow-up time (Table 1).

### 3.2. Hemodynamic Studies

Hemodynamic studies were successfully performed in the OCT-LAR groups at baseline and at the end of follow-up. Results are presented in Table 2. At the end of the follow-up, HVPG was reduced in the OCT-LAR group (15.9 ± 2.4 to 12.8 ± 1.6 mmHg, *P* < 0.05).

### 3.3. Biological Values

The evolution of biological tests over the follow-up period is given in Table 3. No statistical difference was found in the baseline plasma concentrations of ET-1 and URO II between the TIPS and OCT-LAR groups (5.9 ± 0.5 pg/mL versus 6.1 ± 0.3 pg/mL, *P* = 0.25; 2399.9 ± 107.8 pg/mL versus 2453.3 ± 77.8 pg/mL, *P* = 0.18, resp.). A slight decrease in ET-1 and URO II was observed in both groups at the end of follow-up but without statistical difference between them (5.6 ± 0.4 versus 5.7 ± 0.3 pg/mL, *P* = 0.41; 2344.8 ± 91.9 versus 2419.3 ± 103.8 pg/mL, *P* = 0.08, resp.). NO*x* concentrations remained statistically unchanged in the TIPS and OCT-LAR groups both at baseline and last follow-up (64.9 ± 2.7 versus 65.5 ± 2.6 *μ*mol/L, *P* = 0.62; 67.9 ± 2.6 versus 68.5 ± 2.0 *μ*mol/L, *P* = 0.5, resp.).

### 3.4. Live Functions

No statistical difference in baseline liver function was observed between the two groups (Table 4). An acute elevation of hepatobiliary enzymes, including AST and ALT at one week after the TIPS placement, was observed. At the same time point, the two hepatobiliary enzymes were improved in the OCT-LAR treatment group. However, no significant difference was found between the groups (Table 5). Two groups of patients did not show different improvement in liver function, assessed by plasma concentrations of AST, ALT, TB, and PT at the end of follow-up (Table 5).

### 3.5. Treatment Complications

One patient in every group presented minor gastrointestinal bleeding (not life threatening and only melena is manifested which means possible rebleeding) during the follow-up period. The complications of OCT-LAR treatment were minor, consisting of abdominal cramps (two patients), transient diarrhea (three patients), hypoglycemia (one patient), flatulence (two patients), and dizziness (two patients). No patient with OCT-LAR treatment developed gallstones, biliary colic, hypertension, or hepatic encephalopathy. However, one patient who received TIPS placement presented procedure-related complications and required rehospitalization during the follow-up period, and 2 patients who received TIPS placement had developed hepatic encephalopathy (Table 6).

## 4. Discussions

Variceal hemorrhage is the most serious complication of PH, accounting for 17–57% of all deaths in cirrhotic patients [20]. The main therapeutic goal in patients with PH is the prevention of initial and recurrent variceal bleeding. Over the past two decades, numerous advances in medical and procedural treatments have been developed for this purpose. One of the largest changes has been the development of TIPS. At present, TIPS placement is generally considered as a first-line therapy in the definitive treatment of PH concomitant with variceal bleeding [21] or as a bridge to liver transplantation because of its relatively low cost and an easier transplantation process [22]. A study showed that TIPS placement within 72 h after acute bleeding can effectively prevent recurrent variceal bleeding [23]. However, complications occur during or after TIPS placement procedures, influencing its clinical use. Therefore, pharmacological agents that may help to improve the prognosis of patients by successfully controlling the initial variceal bleeding could represent an interesting option for those patients who are not candidates for TIPS.

Somatostatin analogues, such as OCT, remain a good choice for acute variceal hemorrhage episodes in patients with PH [24, 25] and also proved to be an effective method to prevent recurrent bleeding episodes [26] with well-known mechanisms for lowering portal pressure [27]. However, the requirement of three times daily administration renders the method impractical for long-term prophylaxis. The long-acting formulation of OCT, namely, OCT-LAR, can be administered once per month with similar efficacy and safety profile to subcutaneous daily administration [28]. Contraction of the vascular smooth muscle via somatostatin receptors and effects on neurohumoral systems on splanchnic vasculature may explain the hemodynamic changes associated with OCT-LAR [29]. After a single intramuscular injection, concentrations of OCT-LAR reach a plateau at day 14 and remain relatively constant for the following 3 to 4 weeks [30]. Therefore, the approach may represent an attractive option for the long-term therapy of variceal bleeding. Spahr et al. [14] suggested that the prolonged administration of OCT-LAR significantly improves the prognosis in several cirrhosis patients.

The purpose of our study is to decide whether the treatment of OCT-LAR exerts a similar effect in improving the prognosis in patients with PH compared with TIPS. Indeed, the choice of TIPS procedure or OCT-LAR administration was based on a clinician's decision only. All patients presented similar characteristics at baseline, were in a stable clinical condition, and remained so during a mean follow-up of around 80 days regardless of whether belonging to the TIPS or OCT-LAR groups.

We use HVPG as a reliable indicator of portal pressure in our study, as supported by numerous authors [31–33]. We also make it one of the most important indicators to judge the effectiveness of OCT-LAR treatment. HVPG had been measured at baseline and at last follow-up time. Our results showed that mean HVPG decreased from 15.9 ± 2.4 to 12.8 ± 1.6 mmHg after the administration of OCT-LAR, which is a significant improvement with about 20% reduction. We also showed that reduction in HVPG by OCT-LAR precedes an improvement in liver function. We found a decrease in AST, ALT, TB, and prothrombin time after 1 week of OCT-LAR administration. The mechanism by which OCT-LAR stimulates liver function remains unclear but could be due to direct or indirect effects on hepatocytes [34]. On the contrary, TIPS resulted in a transient elevation of hepatobiliary enzymes, especially at an early stage after the procedure with an acute increase in AST and ALT in our study. The transient nature of these alterations may be explained by inevitable liver injury both by direct mechanical insult to the hepatic parenchyma as well as potentially decreased antegrade intrahepatic portal venous flow, arterial perfusional compensation, and resultant perfusional ischemia. It is known that a decrease in portal venous blood flow results in an increase in hepatic arterial flow in a mechanism termed the “hepatic arterial buffer response” [35], a phenomenon that may begin instantly after TIPS placement [36]. Although, the temporal evolution of liver function parameters is decreased at the end of follow-up, the liver injury may have an exaggerated effect on patients with poor liver function, which put them at an irreversible higher risk of early mortality [37].

Intrahepatic vascular resistance is regulated by the balance between vasoactive agents, such as ET-1, and vasorelaxing agents, such as nitric oxide (NO). In a cirrhotic liver, increased levels of ET-1 and decreased levels of NO contribute to the contraction of hepatic stellate cells [38]. Wereszczynka-Siemiatkowska et al. showed that peripheral ET-1 levels reflect the stage of PH and liver insufficiency in cirrhosis [39] and are correlated significantly with liver laboratory parameters and HVPG. In our study, we found that after TIPS placement and OCT-LAR treatment, a slight decrease in ET-1 and an increase in NO*x* levels in hepatic venous blood were observed during follow-up period but without difference. Vascular endothelial cells in an enlarged spleen may be an important source of ET-1; OCT-LAR exerted a similar effect on reducing portal pressure and improved the enlarged spleen compared with TIPS. The increase in NO*x* level may be explained by restoring production when subsequent improvements in sinusoidal circulation may increase shear stress on sinusoidal endothelial cells. Moreover, we also found the same downtrend of URO II. As somatostatin-like peptide, URO II has recently been recognized as the most potent vascular mediator in humans, being a minimum of an order of magnitude more potent than ET-1 [40]. Kemp et al. [41] showed that serum URO II is elevated in patients with chronic liver disease and is associated with the severity of the underlying liver disease and the degree of PH. Together with the results of ET-1, NO*x*, and URO II, OCT-LAR and TIPS placement are suggested to exert a possible similar effect in improving the prognosis of PH.

In our study, treatment complications related with OCT-LAR are similar with that in a recent article aimed at investigating the safety and efficacy of OCT-LAR in patients with PH. The study only reported abdominal cramps, diarrhea, and hypoglycemia, whereas no serious adverse effects were detected during the treatment period [42].

The data provided in this study showed similar incidence of minor bleeding and similar liver function, but more clinical complications in the TIPS group. TIPS is a traumatic procedure, and we intended to find some clues in this study to support our notion that LAR could substitute TIPS in some extend. Though they had a similar incidence of minor bleeding and similar liver function, LAR had less clinical complications than TIPS. Therefore, LAR is preferred in our point of view when a treatment decision is made.

In conclusion, our data showed that the prolonged administration of OCT-LAR presented a virtually similar effect on improving the hemodynamic parameters and liver function in patients with PH compared with TIPS, with no apparent serious adverse effects. The approach is effective in reducing the possibility of PH-related recurrent variceal bleeding in patients with advanced PH. However, owing to the retrospective nature of the approach, further studies on long-term treatment with OCT-LAR in larger groups of patients are still needed.

## Figures and Tables

**Table 1:** Patient characteristics.

	TIPS	OCT-LAR	*P*
Patients (M/F)	9/3	8/4	0.65
Age (years)	49.6 ± 8.7	50.8 ± 9.8	0.76
Child scores	8.1 ± 0.5	8.0 ± 0.6	0.72
Chronic active hepatitis/alcoholic cirrhosis	9/3	9/3	1.0
Follow-up time (d)	79.2 ± 19.9	82.1 ± 16.8	0.69

**Table 2:** Hemodynamic values at baseline and last follow-up.

OCT-LAR (*N* = 12)	Baseline	Last follow-up	*P*
WHVP (mmHg)	26.9 ± 2.3	22.6 ± 1.6	<0.05
FHVP (mmHg)	11.0 ± 1.1	9.8 ± 1.3	<0.05
HVPG (mmHg)	15.9 ± 2.4	12.8 ± 1.6	<0.05

**Table 3:** Biological values at baseline and last follow-up.

	TIPS (*N* = 12)	OCT-LAR (*N* = 12)	*P* value
*Baseline*			
ET-1 (pg/mL)	5.9 ± 0.5	6.1 ± 0.3	0.25
URO II (pg/mL)	2399.9 ± 107.8	2453.3 ± 77.8	0.18
NO*x* (*μ*mol/L)	64.9 ± 2.7	65.5 ± 2.6	0.62
*Last follow-up*			
ET-1 (pg/mL)	5.6 ± 0.4	5.7 ± 0.3	0.41
URO II (pg/mL)	2344.8 ± 91.9	2419.3 ± 103.8	0.08
NO*x* (*μ*mol/L)	67.9 ± 2.6	68.5 ± 2.0	0.50

**Table 4:** Live functions at baseline.

	TIPS (*N* = 12)	OCT-LAR (*N* = 12)	*P*
AST (U/L)	94.6 ± 49.3	97.2 ± 37.3	0.89
ALT (U/L)	101.3 ± 57.2	100.8 ± 35.3	0.98
TB (*μ*mol/L)	64.7 ± 16.9	51.9 ± 16.3	0.07
Prothrombin time (s)	17.6 ± 1.3	18.1 ± 1.8	0.51

**Table 5:** Live functions at one week and last follow-up.

	TIPS (*N* = 12)	OCT-LAR (*N* = 12)	*P*
*One week*			
AST (U/L)	101.8 ± 49.2	90.1 ± 40.6	0.53
ALT (U/L)	104.8 ± 48.1	99.6 ± 50.6	0.80
TB (*μ*mol/L)	53.4 ± 11.2	48.6 ± 11.6	0.34
PT (s)	17.5 ± 1.3	17.8 ± 2.1	0.63
*Last follow-up*			
AST (U/L)	85.8 ± 44.6	81.8 ± 41.6	0.82
ALT (U/L)	91.8 ± 49.5	80.3 ± 38.2	0.53
TB (*μ*mol/L)	45.4 ± 11.7	42.1 ± 9.7	0.47
PT (s)	16.1 ± 1.0	17.0 ± 1.7	0.13

**Table 6:** Treatment complications.

	TIPS (*N* = 12)	OCT-LAR (*N* = 12)
Rebleeding	1	1
Deaths	0	0
Serious^∗^	1	0
Abdominal cramps	0	2
Diarrhea	0	3
Hypoglycemia	0	1
Constipation	0	0
Flatulence	0	2
Cholelithiasis/biliary colic	0	0
Hypertension	0	0
Dizziness	0	2
Hepatic encephalopathy	2	0

^∗^Serious means requiring hospitalization, fatally life threatening, significantly incapacitating, or requiring medical intervention.
